# Colopharyngoplasty in Patients with Severe Pharyngoesophageal Corrosive Injury: A Complicated but Worthwhile Procedure to Restore GI Tract Continuity, A Case Series

**Published:** 2017

**Authors:** Mahdi Zangi, Seyed Reza Saghebi, Ali Biharas Monfared, Seyedamirmohammad Lajevardi, Mohammad Behgam Shadmehr

**Affiliations:** 1 Tracheal Diseases Research Center, National Research Institute of Tuberculosis and Lung Diseases (NRITLD), Shahid Beheshti University of Medical Sciences, Tehran, Iran; 2 Lung Transplantation Research Center, National Research Institute of Tuberculosis and Lung Diseases (NRITLD), Shahid Beheshti University of Medical Sciences, Tehran, Iran; 3 Faculty of Health, York University, Toronto, Canada.

**Keywords:** Colopharyngoplasty, Pharyngoesophageal stricture (PES), Corrosive injury, Caustic injury

## Abstract

**Background:**

Pharyngoesophageal strictures (PES) after corrosive injury impose a problematic condition for both physicians and patients in terms of their management and patients’ quality of life. Colopharyngoplasty is a complex procedure, which is used to restore swallowing in these severely disabled patients. We describe our experience in treating nine patients with severe PES after corrosive injuries in a referral center.

**Materials and Methods:**

A retrospective analysis of our database from 2009 to 2014 showed nine patients (seven men; age range: 18 to 47 years) with severe PES who underwent colopharyngoplasty ∼6 months (range: 4–10) after caustic material ingestion. All patients had a feeding jejunostomy tube before reconstruction. Esophagectomy with or without gastrectomy was performed in all patients, except for one; thereafter, an isoperistaltic segment of the left colon was pulled up, and a pharyngocolic anastomosis was performed. Eight patients had a tracheostomy created either before reconstruction due to respiratory symptoms or at the time of definitive surgery to prevent aspiration in the early post-operative period.

**Results:**

Almost all survivors had a satisfactory swallowing at the end of the follow-up (range: 4–60 months). The jejunostomy tube could be removed in all of the patients after a median of 5 months. One patient died of sepsis due to graft necrosis in the immediate post-operative period. Another patient died 5 months after the first surgery following a revision surgery for intractable dysphagia. At the end of the follow-up, only one patient tolerated tracheostomy tube decannulation. Two patients required laryngotracheal dissociation because of massive aspiration and recurrent episodes of pneumonia. Five patients still had a tracheostomy because of an severely destroyed larynx (two patients) and aspiration (three patients).

**Conclusion:**

Colopharyngoplasty is considered a complicated but trustworthy procedure to restore gastrointestinal tract continuity after severe corrosive injury. Undeniably, laryngeal involvement adversely affects the functional outcome. The post-operative course is frequently protracted, accompanied with several problems. Aspiration is nearly the most problematic event in the early post-operative period, which mandates a multidisciplinary approach to manage it.

## INTRODUCTION

Among benign causes of upper aero-digestive tract stenosis, caustic injuries are one of the most problematic situations to deal with. The management of pharyngolaryngeal involvement after ingestion of corrosive materials is challenging (1). Despite the presence of several therapeutic options, the gold standard has not been introduced yet, and each patient has to be approached individually. Awareness of some critical points (including the site and length of stenosis, status of the larynx and pharynx, and any previous procedure) is of great importance before proceeding to any kind of surgery. Esophageal reconstruction at this level requires a great deal of experience; however, serious complications, unpredictable outcomes, and overall unsatisfactory functional results are common (2). Here, we describe our experience in managing patients with severe pharyngoesophageal strictures (PES) after corrosive injuries in an Iranian referral center, focusing on technical issues and functional outcomes.

## MATERIALS AND METHODS

From January 2009 to January 2014, nine patients with severe PES after ingestion of caustic materials underwent colopharyngoplasty at our referral thoracic surgery ward. Four of them underwent an emergent tracheostomy before admission due to respiratory distress. A protective tracheostomy was performed for the other patients at the time of surgery, except for one patient with an intact larynx.

All patients had a gastrostomy or jejunostomy tube for correction of malnutrition. Two of them underwent esophagectomy at other centers due to severe initial injuries.

The pre-operative evaluation consisted of a comprehensive physical examination, Ear-Throat-Nose (ENT) consultation, and awake laryngoscopy, as well as bronchoscopy and esophagoscopy under general anesthesia. The patients’ psychiatric stability before definitive surgery was assessed and confirmed via psychiatric consultation.

### Surgical technique:

At the initial stage, a midline laparotomy was performed. Our first option for esophageal substitution was the left colon. The continuity of the marginal artery was assessed via a meticulous mobilization of the mesocolon from the sigmoid to the hepatic flexure. The adequacy of the length of the graft was confidently estimated using a silk suture between the mastoid process and xiphoid. It seems that this length could be adequate for any esophageal replacement.

Esophagectomy was performed in six patients (three transhiatal and three transthoracic). In one patient, the native esophagus was not resected. In the other patients who underwent a prior esophagectomy, a retrosternal route was created for the passage of the graft. An oblique incision was created along the anterior border of the sternocleidomastoid muscle.

The side of the neck incision was selected on the basis of the least injured site of the pharynx above the uppermost level of obstruction. With an appropriate pre-operative evaluation for creating a pharyngotomy in the posterior pharyngeal wall, we used the assistant’s index finger into the patient’s mouth and the surgeon palpated its tip.

**Figure 1.**
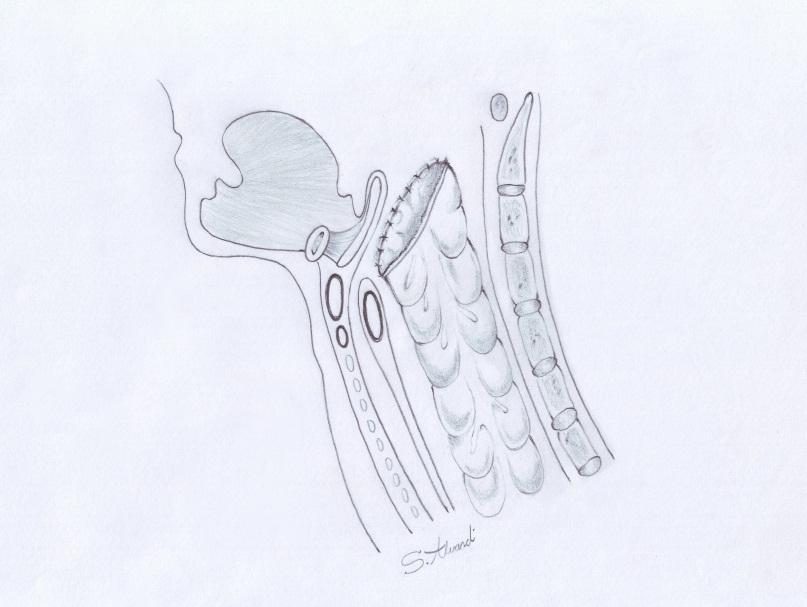
Colopharyngostomy in posterior pharyngeal wall.

In three patients, the thoracic inlet narrowed; thus, it had to be enlarged by resecting the half of the manubrium, medial part of the clavicle, and 1^st^ and 2^nd^ ribs. A supraglottic partial laryngectomy (SPL) was performed in six patients (3). Finally, the colonic graft was passed to the neck through the retrosternal route or posterior mediastinum. Thereafter, an end-to-end pharyngocolic anastomosis was performed using a single layer of interrupted 3-0 vicryl sutures; the distal part of the graft was then anastomosed to the stomach, duodenum, or a Roux-en-Y loop of the jejunum, whichever was the most appropriate and available (Figure 1). The previous jejunostomy tube was used for enteral feeding during the post-operative period.

### Post-Operative Management:

We routinely started enteral nutrition via the jejunostomy tube on the 5 ^th^ post-operative day, except for cases with complications. Simultaneously, we initiated oral feeding if the patients were able to tolerate such. First, a semisolid diet was started to minimize aspiration, which is a common problematic event particularly in the early postoperative period. We expected higher risks of aspiration with fluids. According to our experience on tracheostomy tube as an effective tool to control aspiration, we did not attempt to decannulate such immediately. Thus, the tracheostomy tube was removed only if there was no evidence of aspiration. Two months following oral feeding toleration, the jejunostomy tube was then removed.

### Follow-up:

Before and after the surgery, all patients were educated on how to control aspiration. In these educational sessions, our team trained the patients on special diets and different maneuvers in the post-operative period. After discharge, follow-ups were scheduled every 3 months and 6 months during the first and second years, respectively, and then annually. A good functional outcome was defined as the situation when the patients were able to eat or breathe without the need of any appliance and stoma.

## RESULTS

Table 1 presents the demographic and clinical information of the nine patients. The median age at presentation was 34.3 years (range, 18–47). Seven patients (77.8%) were men. Five patients (55.5%) had ingested alkaline agents, and the remaining patients used acids. Of the nine patients, six (66.7%) had ingested caustic agents accidentally, and the others were hospitalized owing to suicide attempts. The mean duration between the initial injury and the time of reconstruction was 5.94±2.27 months (range: 4–10).

**Table 1.** Clinical characteristics, management, and complications

**Patients**	**Level of Stricture**	**Previous Esophagectomy**	**Caustic Agent**	**Interval Between Injury and Reconstruction (months)**	**Major early Complications (1 month)**	**Major Late Complications (after first month) / Outcome**
7	Base of tongue	Yes	Alkaline	4	**Death** (Sepsis)	-
3	Epiglottis	Yes	Alkaline	7	Aspiration	Withdrawal of Tracheostomy and jejunostomy tube
4	Epiglottis	No	Alkaline	5	Aspiration Dysphagia	**Death** (18 months due to unknown cause) Withdrawal of jejunostomy tube/ unable to remove Tracheostomy
2	Epiglottis^*^	No	Acid	10	Aspiration Dysphagia	Recurrent pneumonia laryngotracheal dissociation 2 years after operation Withdrawal of jejunostomy tube / Permanent Tracheostomy
8	Epiglottis	No	Acid	6	Aspiration Left VC paralysis Dysphagia	Recurrent pneumonia laryngotracheal dissociation 1 year after operation Withdrawal of jejunostomy tube/ PermanentTracheostomy
9	Epiglottis	No	Acid	9	Aspiration Dysphagia	**Death** (Sepsis following reoperation) Unable to remove Tracheostomy and jejunostomy tube
5	Epiglottis	No	Acid	4	Aspiration Right VC paralysis	Withdrawal of jejunostomy tube / unable to remove Tracheostomy
6	Cervical esophagous^**^	No	Alkaline	4.5	-	Withdrawal of jejunostomy tube
1	Cervical esophagous	No	Alkaline	4	Aspiration	Withdrawal of jejunostomy tube / unable to remove Tracheostomy

* Left vocal cord paralysis before the reconstruction

** No need for Tracheostomy

**Figure 2.**
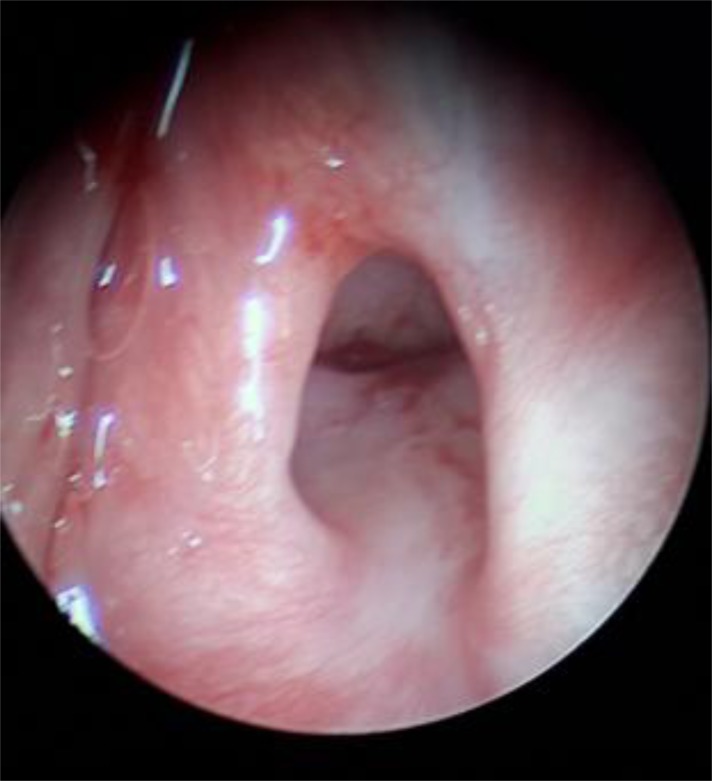
Severe adhesion of epiglottis to posterior pharyngeal wall

As shown in Figure 2, the epiglottis was firmly attached to the posterior pharyngeal wall in six patients (66.7%), which made the detailed assessment of the larynx impossible without a flexible laryngoscopy/bronchoscopy. The assessment showed a severe laryngopharyngeal involvement after the caustic injury. Based on the ENT consultation, one of them had a left vocal cord dysfunction before surgery. All of these patients underwent a supraglottic laryngectomy with accompanying removal of the deformed epiglottis. In one patient, the level of obstruction was higher, involving the base of the tongue, which necessitated a resection of the central portion of the hyoid bone (suprahyoid pharyngectomy).

One patient died of sepsis and multi-organ failure on the 3^rd^ post-operative day due to graft necrosis.

The most common complications in the early post-operative period were aspiration (seven patients, 77.8%) and dysphagia (four patients, 44.4%). Indeed, all survivors had different amounts of aspiration fluid in the postoperative period, except for one patient with an intact larynx pre-operatively.

Although the aspiration was diminished via the rehabilitative swallowing program during the follow-up period, it has still remained to some degree in five patients whose tracheostomy tube cannot be withdrawn yet. Only one patient tolerated the removal of both the tracheostomy and jejunostomy tubes safely at the end of the follow-up period.

Two patients with dysphagia responded well to dilatation with a near-normal swallowing thereafter. In another patient, dysphagia resolved after a second surgery for revision of anastomosis by releasing the colonic graft only in the neck and performing a new oropharyngeal anastomosis. A jejunal free flap was also created for the last patient with persistent dysphagia, despite frequent dilatations; unfortunately, he died of sepsis due to graft necrosis.

There were two cases of vocal cord paralysis after postoperative laryngoscopy. Recurrent episodes of aspiration pneumonia necessitated laryngotracheal dissociation in two patients (one patient with vocal cord dysfunction preoperatively and one with vocal cord paralysis postoperatively).

At the end of the follow-up period, all survivors (six patients) had a satisfactory swallowing. The jejunostomy tubes were removed ∼2 months after oral feeding toleration.

## DISCUSSION

The actual incidence of caustic injuries in the upper aero-digestive tract is underestimated in developing countries owing to cultural, social, and economic factors (4). The management of patients with caustic injury is generally a demanding issue, particularly in adults with suicidal attempts as the most common cause of injury (5). Consequently, these patients are not as cooperative as others. Psychiatric stability is an important prerequisite before any reconstruction in these patients.

The incidence of pharyngeal and upper respiratory tract involvements following caustic ingestion has been rarely reported (6); decision-making for these injuries is still problematic both for physicians and patients. Different types of stomas (such as tracheostomy, jejunostomy, and esophagostomy) and inability to breathe and swallow normally lead to various degrees of physical, social, and psychological difficulties. Getting rid of all of these difficulties seems unachievable at least in the early postoperative period.

Even though stridor and saliva drooling are high-specific symptoms for severe caustic esophageal injuries (7), their absence does not rule out pharyngeal/laryngeal involvement. In severe esophageal injuries after caustic ingestion, concomitant pharyngeal/laryngeal injuries may be misdiagnosed (8). Therefore, a detailed pharyngolaryngeal examination is mandatory immediately after the first visit and before esophageal resection and any kind of gastrointestinal (GI) reconstruction.

The signs of lower respiratory tract involvement, including wheezing, tachypnea, and oxygen requirement, are rarely observed owing to the protective mechanism of the glottis for aspiration (9). This mechanism operates at the cost of unpredictable damage to the laryngeal structures.

Management of concurrent esophageal and pharyngolaryngeal involvements after caustic injuries requires experience and awareness of different therapeutic options (10). If it fails to achieve satisfactory results after the first surgery, it would be too difficult to achieve such in the latter surgeries but is still possible when performed by experienced surgeons (11).

Some critical points have to be considered before proceeding to any kind of reconstruction for severe corrosive GI injuries: the site of the stricture, length of the stricture, status of the larynx and pharynx, function of the vocal cords, time interval between the caustic injury and presentation, existence of tracheostomy before reconstruction, previous esophagectomy, pre-operative nutritional condition, and psychiatric status.

The site of the stricture has to be clearly defined before reconstruction. Hypopharyngeal and upper esophageal sphincter strictures may not be easily evident in esophagography, indicating the importance of a direct examination of the pharynx and cervical esophagus via endoscopy. Otherwise, an esophagocolic anastomosis may fail as a result of an undiagnosed pharyngeal injury.

Some authors proposed that the requirement for an emergent esophageal resection could adversely affect the survival and quality of life of patients (4,12). Most of these emergent surgeries will result in esophagectomy, cervical esophagostomy, frequent concurrent gastrectomy (13), and consequently the need for a complex reconstructive procedure in the future. Unfortunately, some of these emergent resections are extremely aggressive and unnecessary while only relying on immediate endoscopic findings (14). Hence, the decision to perform an emergent surgery for corrosive injuries has to be made judiciously, mostly on the basis of clinical findings rather than radiologic or endoscopic findings alone.

In general, strictures following corrosive injuries tend to be long, narrow, rigid, and multiple (10). Thus, it is comprehensible that dilatations, as an initial therapeutic option (15), accompany a high failure rate. In short segment stenoses, a free jejunal graft or forearm flap could be appropriate for reconstruction (16,17).

In long segment strictures involving the cervicothoracic esophagus besides the pharynx or a history of esophagectomy, there is no other option but to use a visceral graft (stomach or colon) for esophageal replacement. The partial or total damage to the stomach by corrosive agents makes it an unfitting esophageal substitute in many cases (1). The long length and excellent blood supply of the colon have made it a superior esophageal conduit to other substitutes particularly for high-level pharyngeal stenoses (18,19). Both the right and left colon could be used for this purpose provided that the vascularization of the colonic segment is adequate. The viability of the colonic graft has to be confirmed before its division. We are in favor of an isoperistaltic left colic segment based on the ascending branch of the left colic artery because of its more constant blood supply and better propulsive capacity (20). Careful mobilization of the entire mesocolon from the sigmoid to the hepatic flexure is of paramount importance to visualize the marginal artery in its full length. After several minutes of temporary clamping of the vessels that are to be divided, the small vessels adjacent to the colonic wall have to pulsate visibly. This maneuver might be the simplest and most reliable method of selecting a colic segment (18). We did not perform pre-operative angiography routinely as proposed by some studies to reduce the ischemic complication of colonic grafts (19,21, 22). Most of these ischemic consequences seem to be related to poor venous drainage rather than to the arterial supply; thus, it may not be recognized in routine angiography (23).

The harvested colonic graft could be transferred to the neck via three routes: posterior mediastinal, retrosternal, and subcutaneous. Among them, the posterior mediastinal route is the shortest but is not feasible in patients with a history of esophagectomy. The subcutaneous route is the longest, which makes it the last option.

The uppermost level of the stenosis is another important factor that determines the level of the cervical anastomosis. It has been shown that the results of anastomosis in healthy cervical esophagus are more favorable than those of anastomosis to the hypopharynx (1). It seems that by increasing the level of stenosis from the cervical esophagus to the supraglottic region, the functional outcome becomes poorer after reconstruction. Not only the requirement for a few centimeters longer esophageal substitute but also the interference with laryngeal function and greater risks of aspiration adversely affect the outcomes.

Most patients with laryngeal involvement require a tracheostomy to breathe normally and to prevent aspiration. While tracheostomy appears to be life-saving, an adverse effect on survival has been introduced (24). In our series, we were able to remove both the jejunostomy and tracheostomy tubes in only one patient (good functional outcome). In the other survivors, aspiration was problematic enough to set a limit for tracheostomy extraction. Therefore, unsatisfactory results are common following colopharyngoplasty because of aspiration and difficulty in swallowing in the early post-operative period. Restoration of a near-normal swallowing without aspiration appears too difficult and time-consuming, particularly with increasing levels of stenosis. These patients need a long-term multidisciplinary training program for rehabilitation of swallowing.

In severe cases with obliteration of the piriform sinuses and epiglottic adhesions, it is recommended to excise all scar tissues after pharyngotomy to prevent recurrence of adhesions between the epiglottis and posterior pharyngeal wall (25). There may be a need to resect the superior part of the thyroid ala besides the deformed epiglottis in case of a supraglottic stenosis (SPL) or resection of the base of the tongue (suprahyoid pharyngectomy) (8). This is where preserving the recurrent laryngeal nerves during neck exploration becomes important; otherwise, the tracheostomy will be permanent. Our results (two cases of vocal cord paralysis) support this remark. Both cases required laryngotracheal dissociation due to recurrent episodes of aspiration pneumonia, which could not be prevented by tracheostomy alone.

Dysphagia is another frequent important morbidity following colopharyngoplasty, which could occur as a result of anastomotic stenosis or intrathoracic colic graft redundancy. Four patients in the present study experienced dysphagia after reconstruction. Two of them responded well to endoscopic dilatation. Pharyngocolostomy stenosis necessitated surgical revision in the other two patients. The last but not the least is the importance of the appropriate timing for reconstruction after caustic injury. Most authors have emphasized a minimum of 6 months between the corrosive material ingestion and reconstructive surgery (1,8, 24). This interval seems reasonable to allow the corrosive lesions to heal and mature, minimizing the risk of recurrence at the site of anastomosis.

## CONCLUSION

Colopharyngoplasty is a complicated surgical procedure used for the management of severe PES after corrosive injury. The success of this surgery depends on the awareness of several important factors besides a considerable expertise. A thorough pre-operative evaluation of the pharynx and larynx is mandatory to achieve satisfactory results. Inevitably, laryngeal involvement adversely affects the surgical outcome. Whether early ENT interventions might prevent the formation of irreversible laryngeal strictures is a matter of debate and needs to be further investigated. The postoperative course is generally protracted and complicated by serious morbidities, indicating the need for a strong psychological support from the patients’ family and medical team.
